# XTHs from *Fragaria vesca*: genomic structure and transcriptomic analysis in ripening fruit and other tissues

**DOI:** 10.1186/s12864-017-4255-8

**Published:** 2017-11-07

**Authors:** María Cecilia Opazo, Rodrigo Lizana, Yazmina Stappung, Thomas M. Davis, Raúl Herrera, María Alejandra Moya-León

**Affiliations:** 1 Laboratorio de Fisiología Vegetal y Genética Molecular, Instituto de Ciencias Biológicas, Universidad de Talca, Talca, Chile; 2 Department of Biological Sciences, University of New Hampshire, Durham, NH USA; 3 Present address: Laboratorio de Biología Celular y Farmacología, Facultad de Ciencias Biológicas, Universidad Andrés Bello, Santiago, Chile

**Keywords:** Cell wall disassembling, *Fragaria vesca*, Fruit ripening, Fruit softening, qPCR analysis, Xyloglucan endotransglycosylase/hydrolase, *XTH* gene family, Woodland strawberry

## Abstract

**Background:**

*Fragaria vesca* or ‘woodland strawberry’ has emerged as an attractive model for the study of ripening of non-climacteric fruit. It has several advantages, such as its small genome and its diploidy. The recent availability of the complete sequence of its genome opens the possibility for further analysis and its use as a reference species. Fruit softening is a physiological event and involves many biochemical changes that take place at the final stages of fruit development; among them, the remodeling of cell walls by the action of a set of enzymes. Xyloglucan endotransglycosylase/hydrolase (XTH) is a cell wall-associated enzyme, which is encoded by a multigene family. Its action modifies the structure of xyloglucans, a diverse group of polysaccharides that crosslink with cellulose microfibrills, affecting therefore the functional structure of the cell wall. The aim of this work is to identify the XTH-encoding genes present in *F. vesca* and to determine its transcription level in ripening fruit.

**Results:**

The search resulted in identification of 26 XTH-encoding genes named as *FvXTH*s. Genetic structure and phylogenetic analyses were performed allowing the classification of *FvXTH* genes into three phylogenetic groups: 17 in group I/II, 2 in group IIIA and 4 in group IIIB. Two sequences were included into the ancestral group. Through a comparative analysis, characteristic structural protein domains were found in FvXTH protein sequences. In complement, expression analyses of *FvXTHs* by qPCR were performed in fruit at different developmental and ripening stages, as well as, in other tissues. The results showed a diverse expression pattern of *FvXTH*s in several tissues, although most of them are highly expressed in roots. Their expression patterns are not related to their respective phylogenetic groups. In addition, most *FvXTH*s are expressed in ripe fruit, and interestingly, some of them (*FvXTH 18* and *20*, belonging to phylogenic group I/II, and *FvXTH 25* and *26* to group IIIB) display an increasing expression pattern as the fruit ripens.

**Conclusion:**

A discrete group of *FvXTHs* (*18, 20*, *25* and *26)* increases their expression during softening of *F. vesca* fruit, and could take part in cell wall remodeling required for softening in collaboration with other cell wall degrading enzymes.

**Electronic supplementary material:**

The online version of this article (10.1186/s12864-017-4255-8) contains supplementary material, which is available to authorized users.

## Background

Fruit ripening is a differentiation process that involves several biochemical and biophysical modifications, which contribute to the formation of an attractive fruit for the final consumer or to encourage its seed dispersal [1]. Ripening has been well characterized in climacteric fruits such as tomato, where ethylene induces and controls the associated changes, which are due to the coordinated action of thousands of genes [2]. In contrast, certain fruits such as grape, citrus and strawberries do not display an increase in ethylene production rate in association with ripening, and are classified as non-climacteric. However, many of the biochemical modifications of ripening in non-climacteric fruits resemble those of climacteric fruits, but the molecular mechanism of regulation is not fully understood [3–6].

The cultivated strawberry, *Fragaria* x *ananassa*, has been a widely studied subject of non-climacteric fruit ripening [7–9]. Recent studies indicate that, although strawberry fruit ripening is not accompanied by a burst of ethylene activity as seen in climacteric fruit, nevertheless, ethylene plays an important role in its ripening [8, 9]. However, the complex octoploid genome composition of the cultivated strawberry *F*. x *ananassa* complicates molecular dissection of its physiological processes [10], including the role of ethylene in fruit ripening. Thus, the diploid strawberry model system [11] is an enticing alternate system in which to study the molecular mechanisms of fruit ripening.

The strawberry genus *Fragaria* is composed of about 24 species [10], with 11 diploid species including *Fragaria vesca*, also known as the woodland strawberry. The wild forms of *F. vesca*, comprising four subspecies, are widely distributed in the northern hemisphere, while the so-called ‘Alpine’ or ‘semperflorens’ (perpetual flowering) forms of *F. vesca* ssp. *vesca* have been cultivated in Europe for several hundred years [10]. *F. vesca* ssp. *vesca* Alpine accession Hawaii 4 has been developed as an attractive model for genomic and physiological studies because of its small genome (240 Mb), comparatively simple diploid genomic state, high transformation capacity, and short reproductive cycle [12–15]. Moreover, recent sequencing and revised assembly of the *F. vesca* Hawaii 4 genome [11, 16] has transformed it into an attractive system and excellent reference tool for ripening studies in a non-climacteric fruit.

The expression levels of several genes are modulated during fruit development and ripening, particularly those encoding cell wall associated enzymes such as xyloglucan endotransglycosylase/hydrolase (XTH) [17–20]. XTH enzymes have a fundamental role in cell wall loosening through the modification of xyloglucan chains [21, 22]. XTHs have been isolated from several plant species and tissues, and are encoded by multigenic families [23–26]. The number of genes varies among the species: in Arabidopsis 33 *XTH* gene family members have been described [23], 41 in poplar [27] and 29 in rice [28]. Phylogenetic analysis of these family members allows their assignment into three groups (I/II, IIIA and IIIB) according to the most recent classification [29]. XTH proteins present a *β-jellyroll* secondary structure characteristic of the GH16 (Glycosyl hydrolases) family, with cysteine residues stabilizing the C-terminal and a N-glycosylation site necessary for protein stability [30]. XTH catalyzes hydrolytic (E.C.3.2.1.151) and/or transglycosylation reactions (E.C.2.4.1.207). It has been noticed that several structural characteristics are functionally determinant for each enzyme activity. In this sense, based on the structures of TmNXG1 [31], a predominant endo-xyloglucanase that can also perform xyloglucan endo-transglycosylation at elevated concentration of acceptor substrates and belonging to group IIIA, and PttXET16–34 [32], a transglycosylase and member of group I/II, it has been determined that the length (two to three amino acids) of the denominated loop 2 can influence the type of activity of XTH protein members. Baumann et al. [29] developed a TmNXG1 mutant protein by elimination of loop 2 and obtained an incremental gain in transglycosylase activity. Moreover, the analysis of both structures allowed the identification of structural motifs for the prediction of activity. Expression analysis of *XTH* gene family members in *Arabidopsis thaliana* showed that they are expressed in different tissues and with specific expression patterns in response to hormonal stimuli, even though there are several family members which present similar expression patterns [23].

With the aim to clarify the participation of some XTHs in *F. vesca* fruit development and ripening, we performed the bioinformatic identification of *FvXTH* family members through database analysis. We analyzed the structure of each gene, and after decoding its primary protein sequence the prediction of its secondary structure was done for each predicted protein with the aim to identify structural elements related to a possible activity. The expression profile for each identified *XTH* gene was analyzed by qPCR (quantitative PCR) in *F. vesca*’s fruit at different developmental and ripening stages, and some vegetative tissues. Following this strategy, we were able to identify some *FvXTH*s which could be responsible for the cell wall remodeling required for enlargement and softening of *F. vesca* fruit.

## Results

### FvXTHs identification

**Fig. 1:**
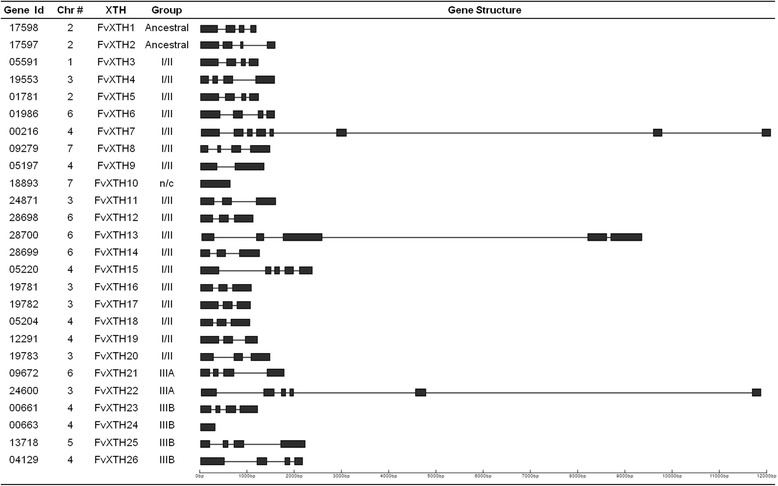
*FvXTH* genes identified in the genome of *F. vesca*. For each gene, the phylogenetic classification according to Baumann [29] is indicated. A diagram of the genic structure of each gene is provided, where bars and thin lines represent coding sequences and introns, respectively. n/c, non-classified

Aiming to identify the complete set of *XTH* family members in *F. vesca*, we searched the public database of the Hawaii 4 strawberry reference genome version 1.1 (https://www.rosaceae.org/species/fragaria/fragaria_vesca/genome_v1.1). Through tblastn analysis (BLAST, Basic Local Alignment Search Tool) we identified 26 *XTH*-like sequences, which are described in Fig. 1. These putative *XTH* genes were named using the nomenclature proposed earlier for *XTH*s [24, 29]. A schematic representation of the structure of each gene is also displayed in the same figure: coding regions are shown as black boxes and introns are shown as thin lines. The genic structure of *FvXTH*s shares similar characteristics regarding the presence of exons and introns, with the exceptions of FvXTH10 and FvXTH24, as shown in Fig. 1. These two last genes showed no introns in their sequences. Interestingly, we found in the FvXTH gene family tandemly organized clusters of two or three family members at four chromosomal locations (Additional file 1: Table S1).

### Phylogenetic analysis

**Fig. 2:**
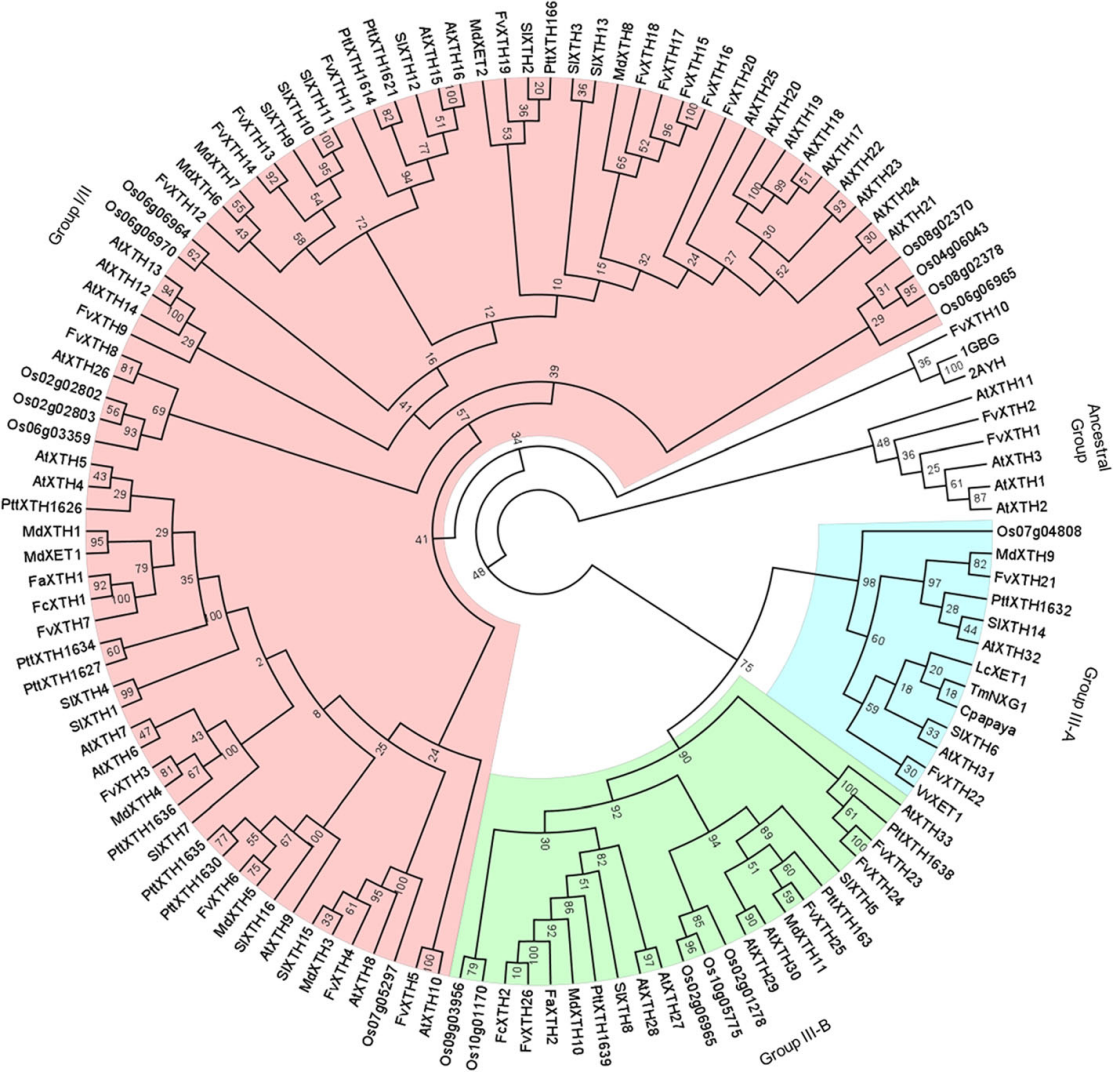
Phylogenetic analysis of FvXTH full-length proteins and other XTH proteins. More than one hundred plant XTH sequences were aligned with the FvXTH protein sequences. The numbers at each fork of the tree indicate the number of times (expressed as percentage) that the group of genes was clustered together in the 100 bootstrap replicates. The numbers of sequences from each taxon were 33 from *Arabidopsis thaliana* (At), 2 from *Fragaria x ananassa* (Fa), 2 from *Fragaria chiloensis* (Fc), 12 from *Malus domestica* (Md), 16 from *Oryza sativa* (Os), 1 from *Carica papaya* (Cp), 16 from *Solanum lycopersicum* (Sl), 1 from *Vitis vinifera* (Vv), 13 from *Populus tremula x Populus tremuloides* (Ptt), 1 from *Populus trichocarpa* (Pt), 1 from *Litchi chinensis* (Lc), 1 from *Tropaeolum majus* (Tm), and 2 bacterial glucanases as external outliers (1GBG, 2AYH). (Genbank accession numbers are listed in Additional file 2: Table S2)

The tree was constructed using ~ 120 XTH amino acid sequences from *Fragaria vesca* and other species, such as *Arabidopsis thaliana*, *Fragaria x ananassa, Fragaria chiloensis*, *Malus domestica*, *Oryza sativa* and *Populus tremula x Populus tremuloides* among others (Fig. 2). The complete list of sequences employed is provided in Additional file 2: Table S2. Two sequences belonging to bacterial glucanases that constitute an ancestral group were included to root the phylogenetic tree. The phylogenetic analysis allows the classification of *FvXTH*s into the three predicted groups according to Baumann [29]. Seventeen *FvXTH* genes were classified into group I/II, 2 into group IIIA and 4 in group IIIB. In addition, two sequences were included into the ancestral group. One gene was grouped as a glucanase (*FvXTH10*) and appears as non-classified (Fig. 1) and its classification as XTH needs to be reviewed.

### Structural element analysis

**Fig. 3:**
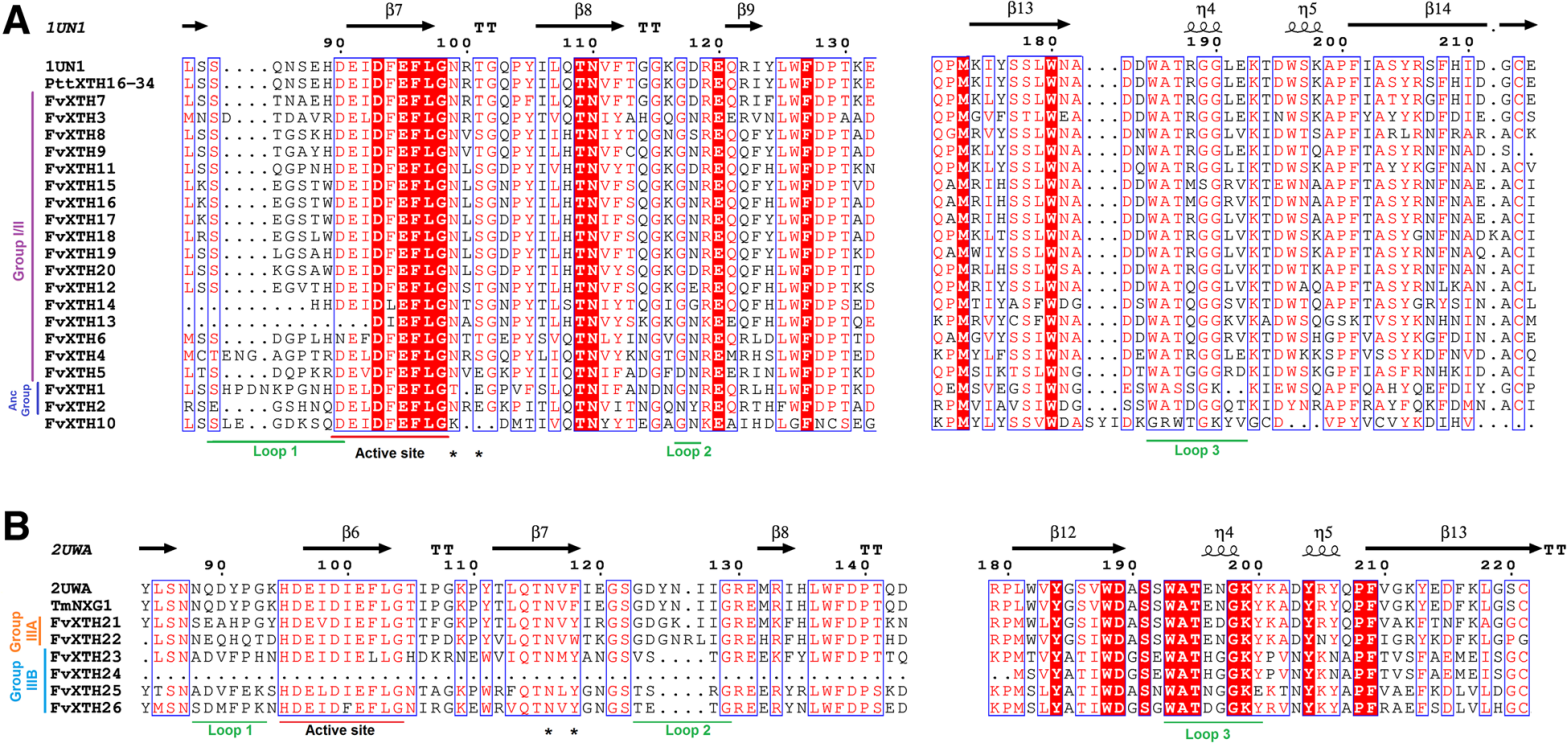
A simplified version of the multiple alignment of deduced amino acid sequences of FvXTHs belonging to group I/II (**a**), and groups IIIA and IIIB (**b**). The catalytic conserved domain (DEIDFEFLG), the secondary structures of β sheets (arrows) and α-helices (spiral), and loops 1, 2 and 3 (lines) are indicated. The conserved residues are shown in red letters; N-glycosylation residues are indicated as *. (The entire alignment is presented in Additional files 3 and 4: Figs. S1 and S2)

The prediction of secondary structures of the *F. vesca’s* XTH protein sequences was obtained using ESPript and the two fully resolved XTH protein structures: PttXET16–34 (PDB id: 1UN1) and TmNXG1 (PDB id: 2UWA). The analysis showed the presence of characteristic domains in the selected sequences (Fig. 3, Additional files 3 and 4: Figs. S1 and S2). The first element identified corresponds to the catalytic domain denoted as D**E**IDF**E**FLG. The first glutamate residue (E) is indicated as the catalytic nucleophile that initiates the enzymatic reaction, and the second E residue functions as a base to activate the entrant substrate. All FvXTH sequences contain both catalytic glutamate residues, except for FvXTH13 that only contains the second E residue and FvXTH24 that lacks most of the important domains of the protein. Figure 3 also shows the presence of the N-glycosylation site denominated as NxT/S/Y (marked with asterisks), that binds N-glycans and is related to protein stability. The presence and location of this N-glycosylation site adjacent to the catalytic domain is characteristic of XTHs from group I/II (Fig. 3A). FvXTH proteins classified within the ancestral group do not display this N-glycosylation site. FvXTH proteins from group IIIB display the characteristic N-glycosylation site displaced around 20 amino acids from the catalytic domain towards the carboxyl terminal, being consistent with other XTHs described from the same group (Fig. 3B). In the case of FvXTH proteins from group IIIA, the N-glycosylation motif is absent in FvXTH22 as it has been reported for other members of this group; nevertheless, this motif apparently exists in FvXTH21 and displaced from the catalytic domain towards the carboxyl terminal (Fig. 3B).

On the other hand, the prediction of secondary structures with β-sheet pattern in all FvXTHs is concordant with the formation of the typical β-jellyroll structure observed in Glycosyl hydrolases (Additional files 3 and 4: Figs. S1 and S2). The presence of an α-helix structure at the carboxyl terminal is also observed. The proteins also contain a conserved domain next to the substrate binding site called as loop 1, loop 2 and loop 3. These loops were identified in FvXTH sequences and are underlined in Fig. 3. The presence and extension of these loops vary among XTH groups. Loops 1 and 3 are in general conserved in extension in FvXTH sequences; major differences are observed in loop 2, as it is shorter in FvXTHs from group I/II and group IIIB compared to group IIIA. The sequence DWATRGG of loop 3 is present in most of FvXTH sequences from group I/II, but not in the ancestral members; this sequence is replaced for SWATEN in FvXTH members from group IIIA.

### Expression analysis of XTH genes identified in *Fragaria vesca*

Structural analysis of FvXTH genes was complemented with the determination of their relative expression analysis by qPCR. The purpose of this analysis was to establish the expression pattern of these genes in fruits at different developmental and ripening stages, as well as, in several vegetative tissues. Twenty-one genes were analyzed using *FvGAPDH* (Glyceraldehyde-3-phosphate dehydrogenase) as normalizer. For genes *FvXTH 1*, *8*, *10*, *15* and *24* it was not possible to design appropriate qPCR primer sets and therefore were not analyzed.

**Fig. 4:**
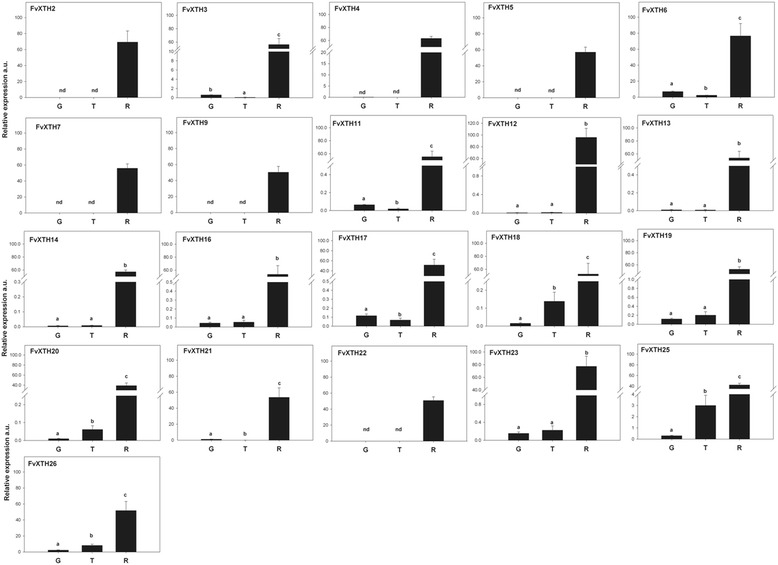
Relative expression levels of *FvXTH*s during development and ripening of *F. vesca* fruit. Each bar represents the relative expression of green (G), transition (T) and ripe fruit (R). Values were normalized against the expression data of FvGAPDH gene and are means ± SE of three independent experiments. Different letters indicate significant differences between fruit stages (*p* ≤ 0.05) according to LSD’s test

Transcripts from genes *FvXTH 3*, *6*, *11*, *12*, *13*, *14*, *16*, *17*, *18*, *19*, *20*, *21*, *23*, *25* and *26* were detected in fruit at all developmental stages, with a substantial increment in expression at the ripe stage (Fig. 4). There is a gradual increment in expression during fruit development and ripening for *FvXTH 18*, *20*, *25* and *26*. On the other hand, transcripts from genes *FvXTH 2*, *4*, *5*, *7*, *9* and *22* were only expressed in fruit at the ripe stage.

The relative expression of FvXTHs in other tissues such as leaves, flowers, runners, stem and roots was also differential (Additional file 5: Fig. S3). All genes accumulate transcripts in the tissues analyzed with the exception of *FvXTH 7, 9, 12, 13, 14, 20* and *22,* which were not detected in runners. For all *FvXTH* genes analyzed the highest expression levels were recorded in roots, except for *FvXTH3* where the same expression level was recorded in roots and stem, and for *FvXTH19* where similar high expression values were recorded in roots and flowers.

### Heat map analysis and gene structure of *FvXTHs*

**Fig. 5:**
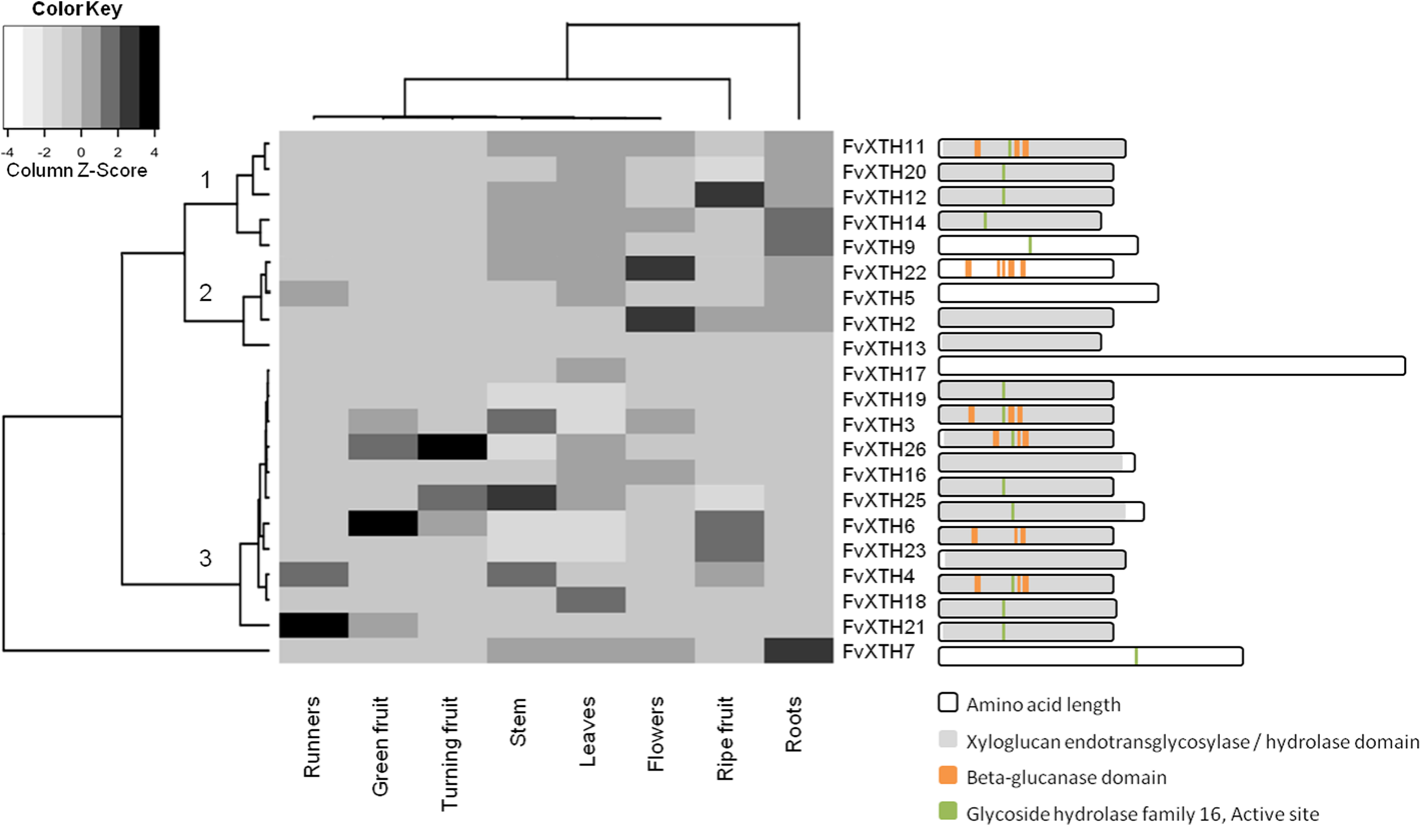
Heatmap of FvXTH transcripts, clustered in groups based of their accumulation profile and tissue specificity. In the left: Heat map clustering. In the right: Description of the different motifs identified in each FvXTH protein sequence by Interproscan. GenBank accession numbers are shown in Fig. 1

The heat map analysis considered 22 *FvXTH* genes and their expression in 5 different tissues and 3 fruit developmental stages. The genes could be divided into three main clusters based on their expression patterns (Fig. 5). Clusters 1 (XTH 9, 11, 12, 14, 20) and 2 (XTH 2, 5, 13, 22) are composed of genes that are highly expressed in roots, with low expression levels in stem, flowers and leaves, and extremely low or undetected transcripts levels in runners. Genes of these clusters also share low expression levels in green and turning fruit stages compared to the high expression level in fruit at the ripe stage (Fig. 4). As mentioned, genes of clusters 1 and 2 share high expression levels in roots, however genes from cluster 1 have higher expression levels than those grouped in cluster 2 (Fig. 5). The remaining genes were grouped into cluster 3, except for *FvXTH7* that was grouped apart, as it is expressed in roots at an extremely high expression level but has no expression at all in runners or in fruit at the immature stages. Cluster 3 genes (XTH 3, 4, 6, 16, 17, 18, 19, 21, 23, 25, 26) displayed their highest expression levels in fruit at the ripe stage, and although they are also expressed in roots their expression levels are lower than in ripe fruit (Fig. 4 and Additional file 5: Fig. S3).

Genes grouped into cluster 1 belong to XTH phylogenetic group I/II, while those clustered in groups 2 and 3 belong to the three XTH phylogenetic groups described, and therefore, no correlation between a phylogenetic group and a specific tissue expression pattern for *XTH* genes in *F. vesca* was evident.

When the XTH predicted protein structures were analyzed using Interproscan, some characteristic domains of XTHs were missing in some of the proteins (Fig. 5). For example, the protein sequences of FvXTH 7, 9, 13 and 22 do not contain the xyloglucan endotransglycosylase/hydrolase domain, and in FvXTH 13 and 22 proteins the β-glucanase domain is also missing. On the other hand, most FvXTHs proteins share in addition to the xyloglucan endotransglycosylase/hydrolase domain others such as the β-glucanase domain and the active site of glycoside hydrolase family 16 domain. The protein sequences of FvXTH 3, 4, 11 and 19, all belonging to XTH phylogenetic group I/II, display the three conserved domains previously mentioned.

## Discussion

A total of 26 putative *XTH* genes were identified in the genome of *F. vesca* (Fig. 1). Our phylogenetic analysis allows the classification of *F. vesca XTH* genes into the three groups currently accepted for the protein family: 17 genes in group I/II, 2 in group IIIA and 4 in group IIIB (Fig. 2). Other two genes were grouped within the ancestral group. Most *FvXTH*s contain several introns which is characteristic of XTH genes [24, 28, 29], with the exception of *FvXTH10* and *FvXTH24*. As shown in Fig. 2, *FvXTH10* was not classified as an XTH. A further analysis of *FvXTH24* indicates that its sequence corresponds to a partial duplication of *FvXTH23,* as exon 4 of *FvXTH23* is identical to the unique exon of *FvXTH24* (Additional file 6: Fig. S4). On the other hand, the genes *FvXTH7*, *FvXTH13* and *FvXTH22* display unusual large introns, and therefore, we cannot exclude a possible mis-assembly of the Hawaii 4 genome.

Gene families arise over evolutionary time through various process of gene duplication and divergence [33]. Duplication events can act on a genomic scale, as in whole genome duplication (WGD) via polyploidization [34], or locally as a consequence of unequal crossing over, transposable element activity, and other forms of local rearrangement that can result in tandem duplication and gene cluster expansion/contraction [33]. An initial survey of gene neighborhoods in the diploid strawberry species, *Fragaria vesca subsp. americana* [35], found that six out of the twenty genes targeted by hybridization probes were tandemly duplicated or clustered, including duplications of the targeted genes ADH (alcohol dehydrogenase), CHS (chalcone synthase), TPS (terpene synthase), and PISTILLATA, and a tandemly duplicated NBS-LRR resistance-like gene. A second targeted NBS-LRR resistance gene was present in four copies, one of which was a pseudogene, within a 20 kb region. Thus, it was not surprising to find that, in the XTH gene family of 26 members, tandemly organized clusters of two or three family members were found at four chromosomal locations.

Gene duplication is commonly followed by structural and/or functional divergence, the latter possibilities including subfunctionalization, neofunctionalization, and pseudogenization [36]. Genes within a local cluster are likely to have arisen from an immediate common ancestor, and to have diverged from each other to a lesser extent than duplicate genes (paralogues) that have become scattered about the genome via processes of chromosome repatterning operating over extended evolutionary periods. Following this pattern, the members of each of the four FvXTH gene clusters we have described tend to occupy terminal or subterminal clades in the phylogenetic tree (Fig. 2).

The prediction of secondary structures in the translated *FvXTH* sequences confirms the existence of the catalytic domain D**E**IDF**E**FLG in all the FvXTH proteins (Fig. 3), except for FvXTH13 that has an incomplete active site and the truncated FvXTH24 sequence. In addition, the N-glycosylation site adjacent to the catalytic domain is present in FvXTHs from group I/II (Fig. 3A), and displaced towards the carboxyl terminal in FvXTHs members of group IIIB (Fig. 3B). The non-existence of this N-glycosylation site in FvXTH22, a member of group IIIA, was also confirmed (Fig. 3B). The prediction of several secondary structures with β-sheet pattern also suggests the formation of the typical β-jellyroll structure observed in Glycosyl hydrolases [37, 38]. Additionally, the existence of the conserved domains named as loops 1, 2 and 3 in all FvXTHs, which are characteristics of this type of protein, affirms its XTH nature.

Important differences in loop 2 are observed between FvXTHs from groups IIIA and IIIB (Fig. 3B). According to Baumann [29] loop 2 interferes with substrate binding. The longer extension of loop 2 in FvXTHs from group IIIA compared to group IIIB has been proposed as a major structural change responsible for its endo-hydrolase activity [29]. In addition, the existence of a volumetric isoleucine in the extended loop 2 of FvXTH IIIA could interfere with the binding to the ligand as it could collide with the glucose unit in the +1 binding site.

Transcriptional analysis of *FvXTH*s in several tissues and in fruit at different development stages followed by heat map analysis allows the grouping of the genes into 3 clusters. Genes belonging to clusters 1 and 2 are highly expressed in roots and in fruit at the ripe stage, while genes of cluster 3 displayed the highest expression level in ripe fruit stage. We found no correlation between the phylogenetic group and a specific tissue expression pattern (cluster). Several XTHs have been described in other plant species and more than one gene has been expressed at the same time in a certain tissue [21, 39, 40]. Interestingly, two or more isoenzymes were co-expressed during development and ripening of several fruit [21, 40].

Moreover, we found the same 26 FvXTH genes differentially expressed in flower and early fruit development stages in the data provided by the work performed by Hollender et al. [14] in *F. vesca*. Although the experimental conditions and tissues analyzed are different, our results concur with the finding that different gene isoforms can be expressed at the same time in a particular tissue.

In strawberry, during the transition from turning to ripe fruit stages, the fruit increases in size (enlargement) and ripens, and at the same time a reduction in fruit firmness is taking place [41]. *FvXTH18* and *FvXTH20*, belonging to phylogenic group I/II, and *FvXTH25* and *FvXTH26* to group IIIB, displayed a substantial increase in their transcription levels as softening is taking place. This suggests a possible role for these genes in cell wall remodeling related to softening of *F. vesca* fruit.

In apple fruit the most abundant transcripts during ripening are those corresponding to *MdXTH2* and *MdXTH10*, whilst *AdXTH4* and *AdXTH5* predominate in ripe kiwifruit [21]. Interestingly, *MdXTH2, AdXTH4* and *AdXTH5* are members of phylogenic group I/II of *XTH*s, similar to the case of *FvXTH18* and *FvXTH20*. Moreover, for *AdXTH*5 and other members of phylogenic group I/II, the respective proteins have xyloglucan endotransglycosylase (XET) activity, which could be involved in fruit softening [21]. On the other hand, *MdXTH10* belongs to group IIIB, as *FvXTH25* and *FvXTH26.* In tomato fruit, the genes *SlXTH5* and *SlXTH8* have been associated with fruit ripening, and both of them belong to group IIIB [42], and SlXTH5 has transglycosylase activity [43]. Members of group IIIA display XEH activity [43–46].

In active developing tissues, such as roots, there is an active transcription of *FvXTH* genes in *F. vesca*. Genes belonging to the different XTH groups are highly expressed. This high expression level in expanding tissues could facilitate the degradation of xyloglucans of the cell wall allowing the rapid wall extension of root tips.

## Conclusions

Some *FvXTH*s belonging to groups I/II and IIIB are preferentially expressed in *F. vesca*’s ripening fruit. Their potential xyloglucan endotransglycosylase activity could be responsible for cell wall remodeling related to the enlargement and softening of *F. vesca* fruit. Finally, this is the first time that the expression pattern of almost all members of the *XTH* multigenic family has been evaluated in a non-climacteric fruit at different developmental stages.

## Methods

### Plant material

*Fragaria vesca* (accession Hawaii 4) seeds were obtained from Macfarlane Greenhouse at New Hampshire University (USA). The seeds were germinated and then sown in pots. Plants were allowed to grow for 6 months in the greenhouse. A set of 30 plants was employed as source of biological material. The fruit was harvested and classified into three developmental and ripening stages according to receptacle size and achene color: green fruit stage (G), corresponding to small fruit with green receptacle and green achenes; turning stage (T), corresponding to fruit with white receptacle and green achenes; and ripe stage (R), corresponding to fully developed fruit with yellow-white receptacle and yellow achenes. Other tissues were obtained from the same plants: runners (Ru), flowers (F), leaves (L), roots (R) and stem (St). Three independent replicates of each fruit stage and vegetative tissues were obtained, and immediately frozen under liquid nitrogen until use.

### Identification of *Fragaria vesca*’s *XTH* genes

The annotated *Fragaria vesca* genome V1.0 (fvesca_v1.0_genemark_hybrid.faa.gz) available at the Genome Database for Rosaceae (GDR) [47] (https://www.rosaceae.org/species/fragaria/fragaria_vesca) was used. Sequences were re-interrogated in *F. vesca* genome V1.1 [11], and annotated sequences were analyzed through BLAST search against the NR (non-redundant) database of NCBI (National Center for Biotechnology Information) as part of the validation procedure. Finally, the identified XTH sequences were mapped onto the *F. vesca* genome V2.0, updating the information and confirming the genetic structure obtained from V1.0. Amino acidic sequences were also obtained using the same approach. Each gene sequence encoding an XTH protein was named as *FvXTH*. The structure of each gene was obtained using *Genome Browser version 2.0* at https://www.rosaceae.org/gb/gbrowse/fragaria_vesca_v2.0.a1/.

### Phylogenetic analysis

Phylogenetic analysis was performed using the deduced amino acid sequences obtained for the *FvXTH* genes identified, and the methodology previously described by Baumann [29]. *Arabidopsis thaliana XTH* sequences available at (https://www.arabidopsis.org/download_files/Proteins/TAIR10_protein_lists/TAIR10_pep_20101214) were used to build the tree, including *XTH* sequences belonging to other organisms and available in public databases. All sequences were obtained from CAZy (The Carbohydrate-Active EnZymes) database (www.cazy.org), and the accession numbers are listed in Additional file 2: Table S2. Briefly, the procedure consisted in the removal of predicted signal peptides through SignalP [48], alignment of sequences using MAFFT [49] and manually refined by using BioEdit (http://www.mbio.ncsu.edu/BioEdit/bioedit.html). The phylogenetic tree was built through PhyML software [50] using maximum likelihood method, using lichenase from *Bacillus licheniformis* (PDB id: 1GBG) and beta-glucanase from *Bacillus amyloliquefaciens*/*Bacillus macerans* (PDB id: 2AYH) as outgroup, tested by bootstrap analysis using 100 resamplings of the data set. The tree was displayed with FigTree software (http://tree.bio.ed.ac.uk/software/figtree/), and the phylogenetic data were deposited in TreeBASE (study identity TB2:S21730).

### Sequence alignment

An alignment analysis of the identified FvXTH sequences was performed with the aim to identify common structural elements present on the sequences encoding putative XTHs in *F. vesca*. For this purpose, the crystal structures of TmNXG1 (PDB id: 2UWA) [29] and that of PttXET16–34 (PDB id: 1UN1) [51] were obtained from the PDB databank (www.pdb.org). Then, by using the online available tool ESPript (http://espript.ibcp.fr/ESPript/ESPript/) [52] the prediction of secondary structures and the presence of structural elements on the FvXTH sequences were obtained.

### qPCR analysis

RNA extractions from fruit and other tissue samples were followed by cDNA (complementary DNA) synthesis as previously reported [20]. qPCR analyses were performed as described in [53]. Primer sequences and efficiency values for each primer pair are shown in Additional file 7: Table S3. Ct (threshold cycle) values were obtained and used to calculate the variations on relative expression levels of the identified *XTH* genes using Pfaffl method [54], and employing *FvGAPDH* as normalizer.

### Heatmap analysis

A color-coded two-dimensional mosaic describing the whole expression matrix (samples vs. gene targets) was built according to [55], in which each tile was colored with a different intensity according to the expression pre-processed data. Gene expression values can be visualized with the colors density ranging from the least (−4) to the most expressed (+4) condition.

### Statistical analysis

Statistical analyses were performed using Statistica v7.0 software. Analysis of variance (ANOVA) was performed and significant differences were determined at *p* ≤ 0.05 using the Scheirer–Ray–Hare test, an extension of the Kruskal–Wallis test, for relative expression analysis.

## Additional files

Additional file 1: Table S1. Information of the genomic sequences of FvXTH genes identified in *F. vesca* genome (DOCX 46 kb)

Additional file 2: Table S2. List of *XTH* sequences employed in the phylogenetic analysis (DOCX 51 kb)

Additional file 3: Figure S1. The multiple alignment of deduced amino acid sequences of FvXTHs belonging to group I/II and ancient group. The catalytic conserved domain (DEIDFEFLG), the secondary structures of β sheets (arrows) and α-helices (spiral), and loops 1, 2 and 3 (lines) are indicated (PDF 1069 kb)

Additional file 4: Figure S2. The multiple alignment of deduced amino acid sequences of FvXTHs belonging to group IIIA and IIIB. The catalytic conserved domain (DEIDFEFLG), the secondary structures of β sheets (arrows) and α-helices (spiral), and loops 1, 2 and 3 (lines) are indicated (PDF 494 kb)

Additional file 5: Figure S3. Relative expression levels of FvXTHs in different *F. vesca* tissues. Each bar represents the relative expression of runners (Ru), flowers (F), leaves (L), roots (R), and stem (St). Values were normalized against the expression data of FvGAPDH gene and are means ± SE of three independent experiments. Different letters indicate significant differences between tissues (*p* ≤ 0.05) according to LSD’s test (PDF 296 kb)

Additional file 6: Figure S4. Comparison of the genomic sequences of FvXTH23 and FvXTH24. At the top: Alignment of the genomic sequences showing the 4 exons of FvXTH23. Exon 4 of FvXTH23 and FvXTH24 share 98.7% sequence similarity. At the bottom: The translated proteins of FvXTH24 and exon 4 of FvXTH23 share the same xyloglucan endo-transglycosylase C–terminus domain according to Pfam analysis (PDF 209 kb)

Additional file 7: Table S3. List of primers employed in the determination of relative expression level of *FvXTH*s from *F. vesca* by qPCR (DOCX 39 kb)

## Abbreviations

ANOVA: analysis of variance
BioEdit: Biological sequence alignment Editor
BLAST: Basic Local Alignment Search Tool
CAZy database: The Carbohydrate-Active EnZymes database
cDNA: complementary DNA
Ct value: threshold cycle value
ESPrint: Easy Sequencing in PostScript
GAPDH: Glyceraldehyde-3-phosphate dehydrogenase
GDR: The Genome Database for Rosaceae
GH: Glycosyl hydrolases
MAFFT: Multiple Alignment using Fast Fourier Transform
NCBI: National Center for Biotechnology Information
NR database: non-redundant database
PCR: polymerase chain reaction
PDB: Protein Data Bank
PhyML: Phylogenetic Maximum Likelihood
qPCR: quantitative PCR
SignalP: prediction of signal peptides
XET: xyloglucan endotransglucosylase activity
XTH: xyloglucan endotransglycosylase/hydrolase
