# Supplementary material for: XTHs from Fragaria vesca: genomic structure and transcriptomic analysis in ripening fruit and other tissues
Source: BMC Genomics. 2017 Nov 7;18:852. doi: 10.1186/s12864-017-4255-8 (PMC5678779; doi:10.1186/s12864-017-4255-8)
Supplement: Supplementary file 1 — Information of the genomic sequences of FvXTH genes identified in F. vesca genome (DOCX 46 kb) [file 12864_2017_4255_MOESM1_ESM.docx]

**Supplementary Table 1.**

Information of the genomic sequences of FvXTH genes identified in *F. vesca* genome.

| **FvXTH** | **Gene Id** | **Chrom #** | **Strand** | **Start** | **Stop** | **Notes** |
| --- | --- | --- | --- | --- | --- | --- |
| 3 | 05591 | 1 | minus | 11,513,266 | 11,512,023 |  |
| 5 | 01781 | 2 | minus | 10,946,663 | 10,945,411 |  |
| 2 | 17597 | 2 | minus | 15,608,294 | 15,606,682 | Genes 17598 and 17597 are a tandemly duplicated pair, in head-to-tail orientation: > > |
| 1 | 17598 | 2 | minus | 15,610,295 | 15,609,092 | Genes 17598 and 17597 are a tandemly duplicated pair, in head-to-tail orientation: > > |
| 20 | 19783 | 3 | plus | 414,630 | 416,126 | Genes 19781, 19782, and 19783 are a tandemly organized three-gene cluster, in the orientation > < > |
| 17 | 19782 | 3 | minus | 417,539 | 416,468 | Genes 19781, 19782, and 19783 are a tandemly organized three-gene cluster, in the orientation > < > |
| 16 | 19781 | 3 | plus | 419,116 | 420,217 | Genes 19781, 19782, and 19783 are a tandemly organized three-gene cluster, in the orientation > < > |
| 4 | 19553 | 3 | plus | 1,307,797 | 1,309,388 |  |
| 11 | 24871 | 3 | plus | 3,709,732 | 3,711,356 |  |
| 22 | 24600 | 3 | minus | 22,988,331 | 22,976,328 |  |
| 19 | 12291 | 4 | minus | 5,314,891 | 5,313,644 |  |
| 9 | 05197 | 4 | minus | 11,812,834 | 11,811,467 |  |
| 18 | 05204 | 4 | plus | 11,889,170 | 11,890,235 |  |
| 15 | 05220 | 4 | minus | 12,060,664 | 12,058,269 | Gene 05220 is closely related to genes 19781, 19782, and 19783, but on a different chromosome and with apparently different intron structure. |
| 7 | 00216 | 4 | minus | 23,760,368 | 23,748,157 |  |
| 26 | 04129 | 4 | minus | 30,047,960 | 30,045,493 |  |
| 23 | 00661 | 4 | plus | 30,912,403 | 30,913,636 | Gene 00661 is a full length version of gene (pseudogene?) 00663. |
| 24 | 00663 | 4 | plus | 30,918,806 | 30,919,123 | Gene 00663 may be an expressed pseudogene copy of gene 00661, in head-to-tail orientation, with a truncated 5' end and an alternate start codon. |
| 25 | 13718 | 5 | plus | 15,195,456 | 15,197,705 |  |
| 21 | 09672 | 6 | plus | 11,690,026 | 11,691,827 |  |
| 12 | 28698 | 6 | plus | 30,240,551 | 30,241,687 | Genes 28698, 28699, and 28700 are a tandemly organized three-gene cluster, in the orientation > > > |
| 14 | 28699 | 6 | plus | 30,244,373 | 30,245,647 | Genes 28698, 28699, and 28700 are a tandemly organized three-gene cluster, in the orientation > > > |
| 13 | 28700 | 6 | plus | 30,247,815 | 30,256,515 | Genes 28698, 28699, and 28700 are a tandemly organized three-gene cluster, in the orientation > > > |
| 6 | 01986 | 6 | minus | 33,273,469 | 33,271,875 |  |
| 8 | 09279 | 7 | plus | 6,818,216 | 6,819,703 |  |
| 10 | 18893 | 7 | minus | 11,456,622 | 11,455,975 |  |
