# Supplementary material for: XTHs from Fragaria vesca: genomic structure and transcriptomic analysis in ripening fruit and other tissues
Source: BMC Genomics. 2017 Nov 7;18:852. doi: 10.1186/s12864-017-4255-8 (PMC5678779; doi:10.1186/s12864-017-4255-8)
Supplement: Supplementary file 2 — List of XTH sequences employed in the phylogenetic analysis (DOCX 51 kb) [file 12864_2017_4255_MOESM2_ESM.docx]

**Supplementary Table 2.**

List of *XTH* sequences employed in the phylogenetic analysis.

| **Organism** | **Name** | **GenBank accession** |
| --- | --- | --- |
| *Arabidopsis thaliana* | At4g13080 (AtXTH1) | AEE83228.1 |
| *Arabidopsis thaliana* | At4g13090 (AtXTH2) | AAY78796.1 |
| *Arabidopsis thaliana* | At3g25050 (AtXTH3) | ABE65966.1 |
| *Arabidopsis thaliana* | At2g06850 (xyloglucan endotransferase EXGT-A1) (AtXTH4) | AAC98464.1 |
| *Arabidopsis thaliana* | At5g13870 (xyloglucan endotransferase EXGT-A4) (AtXTH5) | AAD45126.1 |
| *Arabidopsis thaliana* | At5g65730 (AtXTH6) | AAK73270.1 |
| *Arabidopsis thaliana* | At4g37800 (AtXTH7) | AAM13024.1 |
| *Arabidopsis thaliana* | At1g11545 (AtXTH8) | AAF16642.1 |
| *Arabidopsis thaliana* | At4g03210 (Xth9) (AtXTH9) | AAD14449.1 |
| *Arabidopsis thaliana* | At2g14620 (AtXTH10) | AAC69380.1 |
| *Arabidopsis thaliana* | At3g48580 (AtXTH11) | AAM66971.1 |
| *Arabidopsis thaliana* | At5g57530 (AtXTH12) | AAL15256.1 |
| *Arabidopsis thaliana* | At5g57540 (AtXTH13) | AED96913.1 |
| *Arabidopsis thaliana* | At4g25820 (xyloglucan endotransferase XTR9) (AtXTH14) | AAD12249.1 |
| *Arabidopsis thaliana* | At4g14130 (xyloglucan endotransferase XTR7) (AtXTH15) | AAB18368.1 |
| *Arabidopsis thaliana* | At3g23730 (AtXTH16) | AAM61021.1 |
| *Arabidopsis thaliana* | At1g65310 (Xth-17) (AtXTH17) | AAC27142.1 |
| *Arabidopsis thaliana* | At4g30280 (Xth-18) (AtXTH18) | AAL31883.1 |
| *Arabidopsis thaliana* | At4g30290 (Xth-19) (AtXTH19) | AAK91391.1 |
| *Arabidopsis thaliana* | At5g48070 (Xth-20) (AtXTH20) | AAS77486.1 |
| *Arabidopsis thaliana* | At2g18800 (AtXTH21) | AAD08949.1 |
| *Arabidopsis thaliana* | xyloglucan endotransferase (Xth22; Tch4; At5g57560)  (AtXTH22) | AAA92363.1 |
| *Arabidopsis thaliana* | At4g25810 (xyloglucan endotransferase XTR6) (AtXTH23) | AAB18367.1 |
| *Arabidopsis thaliana* | At4g30270 (xyloglucan endotransferase Meri-5) (AtXTH24) | AAA32828.1 |
| *Arabidopsis thaliana* | At5g57550 (xyloglucan endotransferase XTR3) (AtXTH25) | AAB18364.1 |
| *Arabidopsis thaliana* | At4g28850 (AtXTH26) | AEE85554.1 |
| *Arabidopsis thaliana* | At2g01850 (xyloglucan endotransferase EXGT-A3) (AtXTH27) | AAD21783.1 |
| *Arabidopsis thaliana* | At1g14720 (xyloglucan endotransferase XTR2) (AtXTH28) | AAB18366.1 |
| *Arabidopsis thaliana* | At4g18990 (AtXTH29) | AAM91637.1 |
| *Arabidopsis thaliana* | At1g32170 (XTR4 ) (AtXTH30) | AAB18365.1 |
| *Arabidopsis thaliana* | At3g44990 (xyloglucan endotransferase) (AtXTH31) | AAL07012.1 |
| *Arabidopsis thaliana* | At2g36870 (AtXTH32) | AAD31572.1 |
| *Arabidopsis thaliana* | At1g10550 (AtXTH33) | AAD39577.1 |
| *Carica papaya* | xyloglucan endo-transglycosylase | AAK51119.1 |
| *Fragaria x ananassa* | xyloglucan endotransglucosylase/hydrolase 1 (FaXTH1) | ADE42490.1 |
| *Fragaria x ananassa* | xyloglucan endotransglucosylase/hydrolase 2 (FaXTH2) (fragment) | ADE42491.1 |
| *Fragaria chiloensis* | xyloglucan endotransglucosylase/hydrolase 1 (FcXTH1) | ADE42488.1 |
| *Fragaria chiloensis* | xyloglucan endotransglucosylase/hydrolase 2 (FcXTH2) | ADE42489.1 |
| *Litchi chinensis* | xyloglucan endotransglycosylase 1 | ABK30788.1 |
| *Malus x domestica* | xyloglucan endotransglucosylase/hydrolase 1 | ACD03225.1 |
| *Malus x domestica* | xyloglucan endotransglucosylase/hydrolase 3 | ACD03227.1 |
| *Malus x domestica* | xyloglucan endotransglucosylase/hydrolase 4 | ACD03228.1 |
| *Malus x domestica* | xyloglucan endotransglucosylase/hydrolase 5 | ACD03229.1 |
| *Malus x domestica* | xyloglucan endotransglucosylase/hydrolase 6 | ACD03230.1 |
| *Malus x domestica* | xyloglucan endotransglucosylase/hydrolase 7 | ACD03231.1 |
| *Malus x domestica* | xyloglucan endotransglucosylase/hydrolase 8 | ACD03232.1 |
| *Malus x domestica* | xyloglucan endotransglucosylase/hydrolase 9 | ACD03233.1 |
| *Malus x domestica* | xyloglucan endotransglucosylase/hydrolase 10 | ACD03234.1 |
| *Malus x domestica* | xyloglucan endotransglucosylase/hydrolase 11 (fragment) | ACD03235.1 |
| *Malus x domestica* | xyloglucan endotransglycosylase (Xet01; MdXET1) | AAN07897.1 |
| *Malus x domestica* | xyloglucan endotransglycosylase (Xet02; MdXET2) | AAN07898.1 |
| *Oryza sativa* Japonica group | xyloglucan endotransglycosylase (Os04g0604300) | AAL35903.1 |
| *Oryza sativa* Japonica group | Os10g0577500 | AAL58186.1 |
| Oryza sativa Japonica group | Os10g0117000 | AAL34939.1 |
| *Oryza sativa* Japonica group | Os07g0480800 | BAC45131.1 |
| *Oryza sativa* Japonica group | Os07g0529700 | BAC45142.1 |
| *Oryza sativa* Japonica group | Os08g0237000 (OsXRT5) (OsXTH8) | BAD05469.1 |
| *Oryza sativa* Japonica group | Os08g0237800 | BAD05476.1 |
| *Oryza sativa* Japonica group | Os02g0127800 | BAD07973.1 |
| *Oryza sativa* Japonica group | Os02g0696500 | BAD08162.1 |
| *Oryza sativa* Japonica group | Os09g0395600 | BAD26459.1 |
| *Oryza sativa* Japonica group | Os02g0280200 | BAD28544.1 |
| *Oryza sativa* Japonica group | Os02g0280300 | BAD28545.1 |
| *Oryza sativa* Japonica group | Os06g0696400 | BAD53910.1 |
| *Oryza sativa* Japonica group | Os06g0696500 | BAD53912.1 |
| *Oryza sativa* Japonica group | Os06g0697000 | BAD54452.1 |
| *Oryza sativa* Japonica group | Os06g0335900 | BAD61893.1 |
| *Populus tremula x Populus tremuloides* | xyloglucan endotransglycosylase/hydrolase (XTH3) | ABM91070.1 |
| *Populus tremula x Populus tremuloides* | xyloglucan endotransglycosylase/hydrolase (XTH6) | ABM91066.1 |
| *Populus tremula x Populus tremuloides* | xyloglucan endotransglycosylase/hydrolase (XTH14) | ABM91071.1 |
| *Populus tremula x Populus tremuloides* | xyloglucan endotransglycosylase/hydrolase (XTH21) | ABM91075.1 |
| *Populus tremula x Populus tremuloides* | xyloglucan endotransglycosylase/hydrolase (XTH26) | ABM91063.1 |
| *Populus tremula x Populus tremuloides* | xyloglucan endotransglycosylase/hydrolase (XTH27) | ABM91069.1 |
| *Populus tremula x Populus tremuloides* | xyloglucan endotransglycosylase/hydrolase (XTH30) | ABM91074.1 |
| *Populus tremula x Populus tremuloides* | xyloglucan endotransglycosylase/hydrolase (XTH32) | From ref [29] |
| *Populus tremula x Populus tremuloides* | xyloglucan endotransglycosylase 16A (PttXET16-34; XTH16-34) (XET16A) | AAN87142.1 |
| *Populus tremula x Populus tremuloides* | xyloglucan endotransglycosylase (XTH35; PttXET16-35) | ABL75361.1 |
| *Populus tremula x Populus tremuloides* | xyloglucan endotransglycosylase/hydrolase (XTH36) | ABM91067.1 |
| *Populus tremula x Populus tremuloides* | xyloglucan endotransglycosylase/hydrolase (XTH38) | ABM91064.1 |
| *Populus tremula x Populus tremuloides* | xyloglucan endotransglycosylase/hydrolase (XTH39) | ABM91072.1 |
| *Populus trichocarpa* | xyloglucan endotransglycosylase/hydrolase (XTH27) | ABM91069.1 |
| *Solanum lycopersicum* | xyloglucan endotransglucosylase/hydrolase (XTH1) | BAA03923.1 |
| *Solanum lycopersicum* | xyloglucan endotransglucosylase/hydrolase (XTH2) | AAG00902.1 |
| *Solanum lycopersicum* | xyloglucan endotransglucosylase/hydrolase (XTH3) | AAS46241.1 |
| *Solanum lycopersicum* | xyloglucan endotransglucosylase/hydrolase (XTH4) | AAG43444.1 |
| *Solanum lycopersicum* | xyloglucan endotransglucosylase/hydrolase (XTH5) | AAS46240.1 |
| *Solanum lycopersicum* | xyloglucan endotransglucosylase/hydrolase (XTH6) | AAS46242.1 |
| *Solanum lycopersicum* | xyloglucan endotransglucosylase/hydrolase (XTH7) | AAS46243.1 |
| *Solanum lycopersicum* | xyloglucan endotransglucosylase/hydrolase (XTH8) | BAA88668.1 |
| *Solanum lycopersicum* | xyloglucan endotransglucosylase/hydrolase (XTH9) | AAS46244.1 |
| *Solanum lycopersicum* | xyloglucan endotransglucosylase/hydrolase (XTH10) | NP_001234207.1 |
| *Solanum lycopersicum* | xyloglucan endotransglucosylase/hydrolase (XTH11) | CAA58003.1 |
| *Solanum lycopersicum* | xyloglucan endotransglucosylase/hydrolase (XTH12) | AAF17600.1 |
| *Solanum lycopersicum* | xyloglucan endotransglucosylase/hydrolase (XTH13) | TC160407 |
| *Solanum lycopersicum* | xyloglucan endotransglucosylase/hydrolase (XTH14) | TC164033 |
| *Solanum lycopersicum* | xyloglucan endotransglucosylase/hydrolase (XTH15) | TC163748 |
| *Solanum lycopersicum* | xyloglucan endotransglucosylase/hydrolase (XTH16) | AAZ08349.1 |
| *Tropaeolum majus* | NXG1 (TmNXG1) | CAA48324.1 |
| *Vitis vinifera* | xyloglucan endotransglycosylase 1 | AB074999 |
| *Bacillus licheniformis* | beta-Glucanase | 1GBG |
| Synthetic construct (Bacillus amyloliquefaciens / Bacillus macerans) | 1,3-1,4-beta-D-Glucan 4 Glucano hydrolase (beta-Glucanase, Lichenase) | 2AYH |
