# Supplementary material for: XTHs from Fragaria vesca: genomic structure and transcriptomic analysis in ripening fruit and other tissues
Source: BMC Genomics. 2017 Nov 7;18:852. doi: 10.1186/s12864-017-4255-8 (PMC5678779; doi:10.1186/s12864-017-4255-8)
Supplement: Supplementary file 3 — The multiple alignment of deduced amino acid sequences of FvXTHs belonging to group I/II and ancient group. The catalytic conserved domain (DEIDFEFLG), the secondary structures of β sheets (arrows) and α-helices (spiral), and loops 1, 2 and 3 (lines) are indicated (PDF 1069 kb) [file 12864_2017_4255_MOESM3_ESM.pdf]

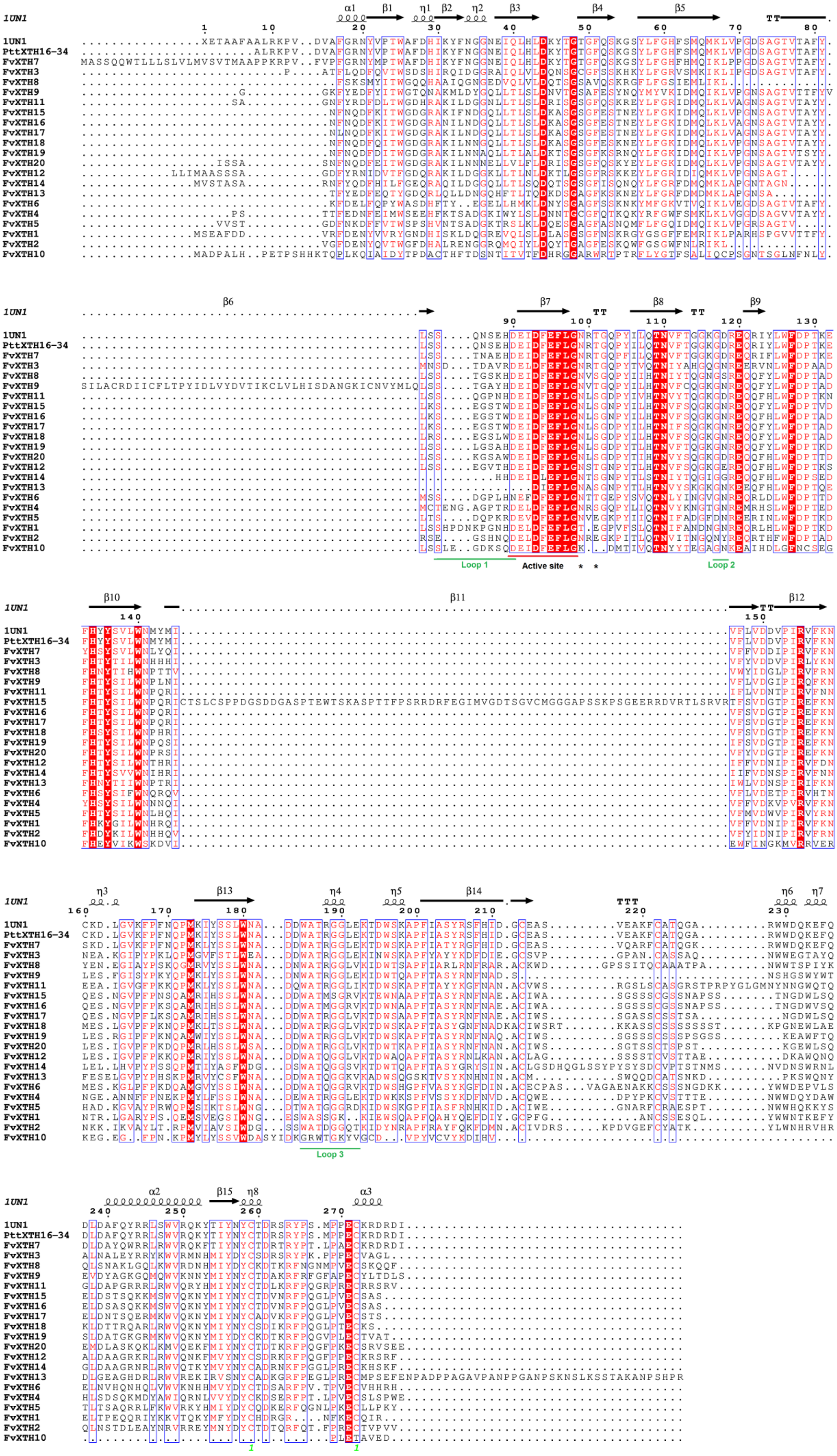
1UN1
PttXTH16-34
FvXTH7
FvXTH3
FvXTH8
FvXTH9
FvXTH11
FvXTH15
FvXTH16
FvXTH17
FvXTH18
FvXTH19
FvXTH20
FvXTH12
FvXTH14
FvXTH13
FvXTH6
FvXTH4
FvXTH5
FvXTH1
FvXTH2
FvXTH10
α1 β1 η1 β2 η2 β3 β4 β5
β6 β7 β8 β9
β10 β11 β12
η3 β13 η4 η5 β14 η6 η7
α2 β15 η8 α3
Loop 1
Active site
Loop 2
Loop 3

Supplementary Figure 1. The multiple alignment of deduced amino acid sequences of FvXTHs belonging to group I/II and ancient group. The catalytic conserved domain (DEIDFEFLG), the secondary structures of β sheets (arrows) and α-helices (spiral), and loops 1, 2 and 3 (lines) are indicated.
