# Supplementary material for: XTHs from Fragaria vesca: genomic structure and transcriptomic analysis in ripening fruit and other tissues
Source: BMC Genomics. 2017 Nov 7;18:852. doi: 10.1186/s12864-017-4255-8 (PMC5678779; doi:10.1186/s12864-017-4255-8)
Supplement: Supplementary file 4 — The multiple alignment of deduced amino acid sequences of FvXTHs belonging to group IIIA and IIIB. The catalytic conserved domain (DEIDFEFLG), the secondary structures of β sheets (arrows) and α-helices (spiral), and loops 1, 2 and 3 (lines) are indicated (PDF 494 kb) [file 12864_2017_4255_MOESM4_ESM.pdf]

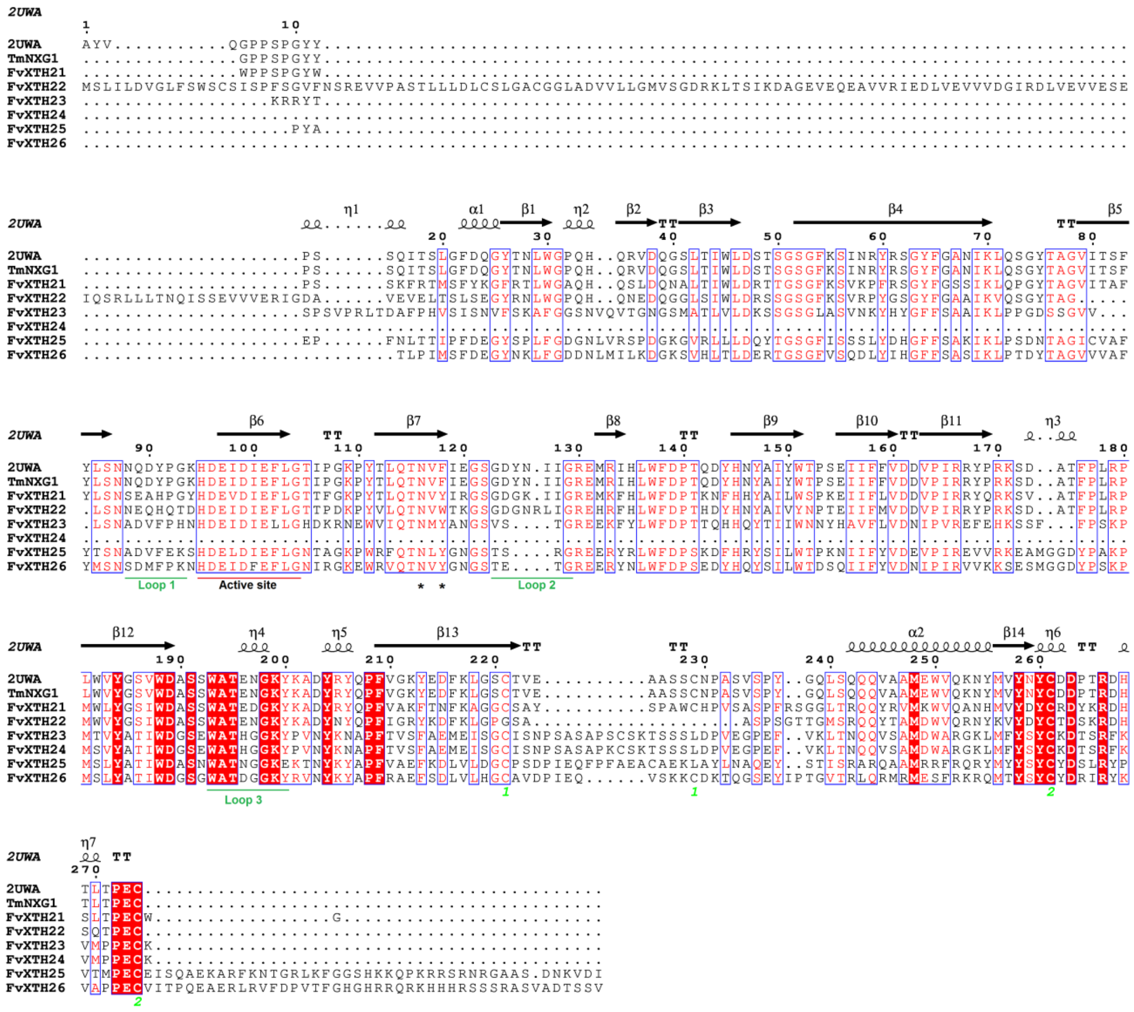

Supplementary Figure 2. The multiple alignment of deduced amino acid sequences of FvXTHs belonging to group IIIA and IIIB. The catalytic conserved domain (DEIDFEFLG), the secondary structures of β sheets (arrows) and α-helices (spiral), and loops 1, 2 and 3 (lines) are indicated.
