# Supplementary material for: XTHs from Fragaria vesca: genomic structure and transcriptomic analysis in ripening fruit and other tissues
Source: BMC Genomics. 2017 Nov 7;18:852. doi: 10.1186/s12864-017-4255-8 (PMC5678779; doi:10.1186/s12864-017-4255-8)
Supplement: Supplementary file 5 — Relative expression levels of FvXTHs in different F. vesca tissues. Each bar represents the relative expression of runners (Ru), flowers (F), leaves (L), roots (R), and stem (St). Values were normalized against the expression data of FvGAPDH gene and are means ± SE of three independent experiments. Different letters indicate significant differences between tissues (p ≤ 0.05) according to LSD’s test (PDF 296 kb) [file 12864_2017_4255_MOESM5_ESM.pdf]

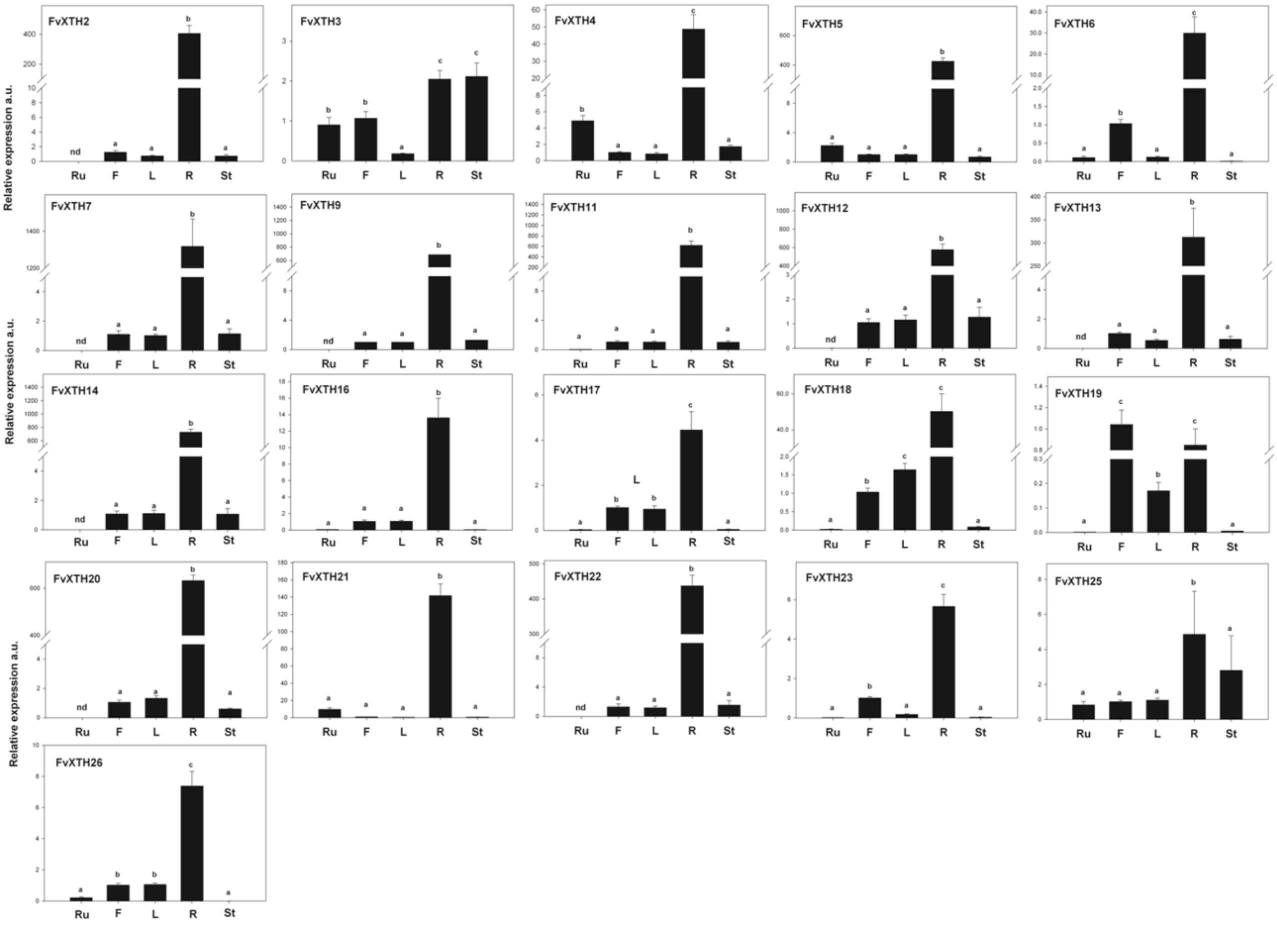

Supplementary Figure 3. Relative expression levels of FvXTHs in different F. vesca tissues. Each bar represents the relative expression of runners (Ru), flowers (F), leaves (L), roots (R), and stem (St). Values were normalized against the expression data of FvGAPDH gene and are means ± SE of three independent experiments. Different letters indicate significant differences between tissues ($p \leq 0.05$) according to LSD's test.
