# Supplementary material for: XTHs from Fragaria vesca: genomic structure and transcriptomic analysis in ripening fruit and other tissues
Source: BMC Genomics. 2017 Nov 7;18:852. doi: 10.1186/s12864-017-4255-8 (PMC5678779; doi:10.1186/s12864-017-4255-8)
Supplement: Supplementary file 6 — Comparison of the genomic sequences of FvXTH23 and FvXTH24. At the top: Alignment of the genomic sequences showing the 4 exons of FvXTH23. Exon 4 of FvXTH23 and FvXTH24 share 98.7% sequence similarity. At the bottom: The translated proteins of FvXTH24 and exon 4 of FvXTH23 share the same xyloglucan endo-transglycosylase C–terminus domain according to Pfam analysis (PDF 209 kb) [file 12864_2017_4255_MOESM6_ESM.pdf]

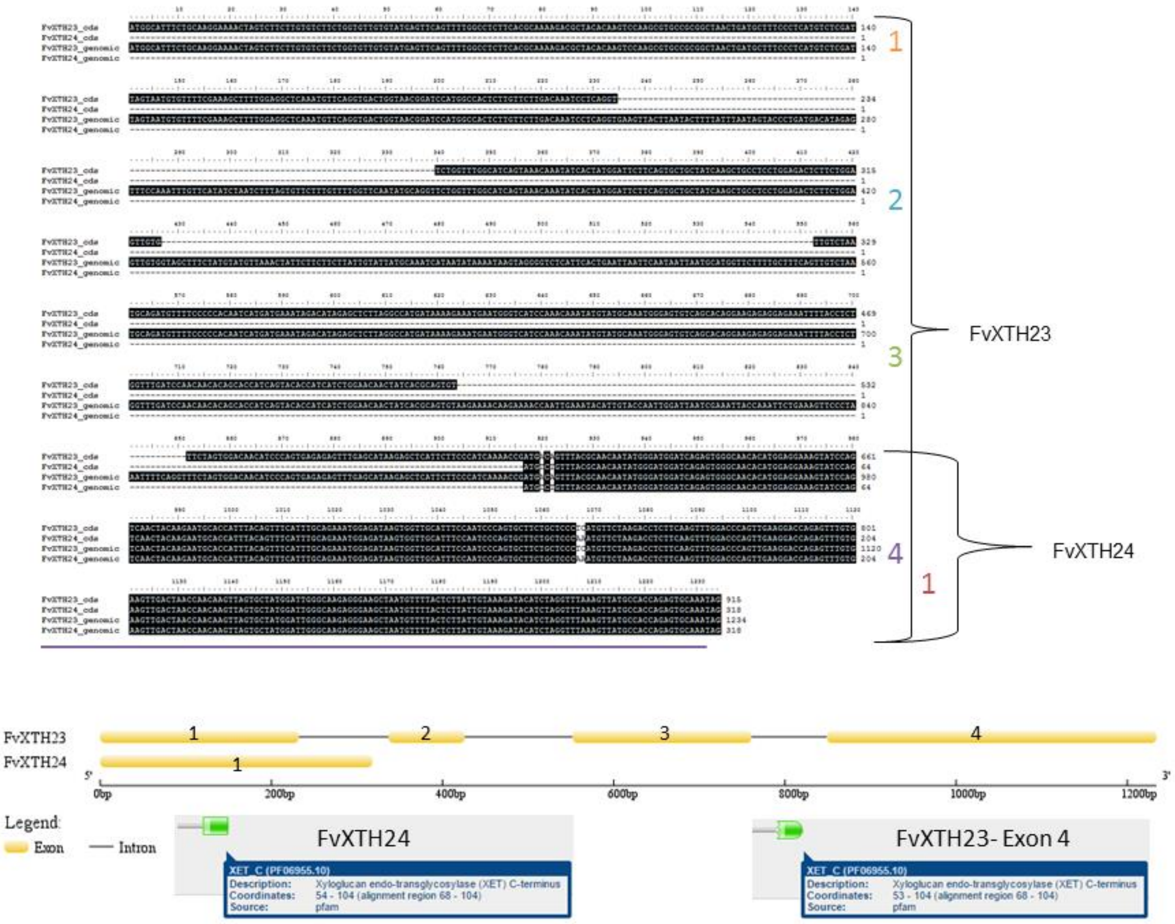

Supplementary Figure 4. Comparison of the genomic sequences of FvXTH23 and FvXTH24. At the top: Alignment of the genomic sequences showing the 4 exons of FvXTH23. Exon 4 of FvXTH23 and FvXTH24 share 98.7 % sequence similarity. At the bottom: The translated proteins of FvXTH24 and exon 4 of FvXTH23 share the same xyloglucan endo-transglycosylase C–terminus domain according to Pfam analysis.
