# Supplementary material for: XTHs from Fragaria vesca: genomic structure and transcriptomic analysis in ripening fruit and other tissues
Source: BMC Genomics. 2017 Nov 7;18:852. doi: 10.1186/s12864-017-4255-8 (PMC5678779; doi:10.1186/s12864-017-4255-8)
Supplement: Supplementary file 7 — List of primers employed in the determination of relative expression level of FvXTHs from F. vesca by qPCR (DOCX 39 kb) [file 12864_2017_4255_MOESM7_ESM.docx]

**Supplementary Table 3.**

List of primers employed in the determination of relative expression level of *FvXTH*s from *F. vesca* by qPCR. Tm of primers is 58°C.

| Primer | Gene ID | Forward sequence (5`→3`) | Reverse sequence (5`→3`) | Amplicon size (bp) | Efficiency (%) |
| --- | --- | --- | --- | --- | --- |
| qFvXTH2 | 17597 | CTATTTTGTGGTGTGATGCTTGC | CATCTGTCTCCCTCCATTCTCAC | 112 | 100 |
| qFvXTH3 | 05591 | ACCAGCCACTTTTCTTCAGGATTT | AGAATTTTGGTCAAGAACGAGCTG | 103 | 94.5 |
| qFvXTH4 | 19553 | AGCTGCACCATCAACTACTTTTG | GTATTGTTGTCGAGCGAGAGGTA | 110 | 100 |
| qFvXTH5 | 01781 | CAGATTACAAGTGGTTCGGTTGT | CGAGCTTCAAACTCCTTGTCTT | 102 | 100 |
| qFvXTH6 | 01986 | GTGTCATTTTGGGTCTCTCATTG | CCTCGTATGTGAAATGGTCAGAA | 102 | 98 |
| qFvXTH7 | 00216 | AGTCTCCAGTCTCCTCCAAACC | GGTGGTTGTGAACATATCTTTGTG | 103 | 99.5 |
| qFvXTH9 | 05197 | CATGATGAGCACTTCCATGGTT | AGAGTCAAGAGCTGCCCATAATC | 110 | 98 |
| qFvXTH11 | 24871 | TTCTGTCTGTCAGTTGTCCCTTC | GAGGTTTCCACCATCGAGTATCT | 113 | 93 |
| qFvXTH12 | 28698 | GCTGGTGATTTCTACCGAAACAT | AGCCAGACCCTAAAGTCTTGTCA | 112 | 97 |
| qFvXTH13 | 28700 | ATGATGGCTAGCATACCTGCTTC | TCCTGTGTGAGTGTGAAGTGTTG | 115 | 99.5 |
| qFvXTH14 | 28699 | AACAACGTGCACAAATACTCGAC | CCGTCCAAATAGGTATTGGTTCT | 104 | 98 |
| qFvXTH16 | 19781 | CTGTTTGTACCATTTCTGCTTGC | GTTGGCCGTCGTTGAGTATATTT | 118 | 96.5 |
| qFvXTH17 | 19782 | TCTGCTGGTAACTTGAACCAAGA | GAGGCTTTATCGAGGGAGAGAGT | 104 | 97.5 |
| qFvXTH18 | 05204 | GCGTCTTTCAAATCTGCTCTTTC | TCCCCATGTAATCTGAAAGTCCT | 105 | 97.5 |
| qFvXTH19 | 12291 | ATGCTCTAGCTTTGGTGATGTTC | CTGCGCGTTATTGAGTATTTTG | 119 | 97.5 |
| qFvXTH20 | 19783 | TAGCAGATACTGCCTGGTGTTTT | CACCCCATGTAATCTCAAAATCC | 107 | 99 |
| qFvXTH21 | 09672 | TGGCTACTGGCCAAGTTCTAAAT | CTGTCAAGCCAGATTGTTAATGC | 116 | 96.5 |
| qFvXTH22 | 24600 | AATCCTCGATGTGGGACTATTCT | CAAAGGTCCAGCAACAATGTACT | 108 | 95 |
| qFvXTH23 | 00661 | TTCTTGTGTCTTCTGGTGTTGTG | AGACATGAGGGAAAGCATCAGTT | 109 | 97 |
| qFvXTH25 | 13718 | GGCTGGTTTCTCTCCTTGTTCT | GAAGAGAGGGCTGTATCCTTCGT | 119 | 97.5 |
| qFvXTH26 | 04129 | CTACTTGGTTGCTTTGGTTGTTC | CCCAAAGAGCTTGTTGTATCCTT | 100 | 99.5 |
| FvGAPDH | 07104 | TCCATCACTGCCACCCAGAAGACTG | AGCAGGCAGAACCTTTCCGACAG | 96 | 93 |
